# A global perspective on evolving bioinformatics and data science training needs

**DOI:** 10.1093/bib/bbx100

**Published:** 2017-08-29

**Authors:** Teresa K Attwood, Sarah Blackford, Michelle D Brazas, Angela Davies, Maria Victoria Schneider

**Affiliations:** 1 University of Manchester, School of Computer Science, Manchester, United Kingdom of Great Britain and Northern Ireland; 2 Lancaster University, Lancaster, United Kingdom of Great Britain and Northern Ireland; 3 Ontario Institute for Cancer Research, Informatics and Bio-computing, 101 College St, Suite 800, Toronto, Ontario Canada; 4 The University of Manchester, School of Biological Sciences, Manchester, United Kingdom of Great Britain and Northern Ireland; 5 University of Melbourne Melbourne Institute, Lab-14, 700 Swanston St, Melbourne Carlton Victoria, Australia

**Keywords:** bioinformatics training, training survey, skills gap, computational and statistical competency, data science, training trainers

## Abstract

Bioinformatics is now intrinsic to life science research, but the past decade has witnessed a continuing deficiency in this essential expertise. Basic data stewardship is still taught relatively rarely in life science education programmes, creating a chasm between theory and practice, and fuelling demand for bioinformatics training across all educational levels and career roles. Concerned by this, surveys have been conducted in recent years to monitor bioinformatics and computational training needs worldwide. This article briefly reviews the principal findings of a number of these studies. We see that there is still a strong appetite for short courses to improve expertise and confidence in data analysis and interpretation; strikingly, however, the most urgent appeal is for bioinformatics to be woven into the fabric of life science degree programmes. Satisfying the relentless training needs of current and future generations of life scientists will require a concerted response from stakeholders across the globe, who need to deliver sustainable solutions capable of both transforming education curricula and cultivating a new cadre of trainer scientists.

## Introduction

In recent decades, relentless technological advances have inexorably changed the practice of life science research. One of the most conspicuous changes is the core role now taken by bioinformatics. Bioinformatics is an intrinsically interdisciplinary and broad-ranging field, harnessing aspects of computer science, mathematics and statistics to store, manage, analyse and interpret biological data—it is fundamentally data-driven and computational. In recent years, the advent of high-throughput instrumentation has placed the growing volumes of biological data in the spotlight, blurring the boundaries between bioinformatics and data science. Thus, as the evolving landscape of life science research has become more data-driven, integrative and computational, the need for biomedical scientists to acquire ‘bioinformatics’ skills (in the broadest sense) has grown. Of course, not every research scientist must become a bioinformatician; however, acquiring at least a minimum level of computational skills can help life and computational scientists to communicate and interact with one another more effectively, and improve critical thinking about research findings [1, 2]. Even experienced bioinformaticians—many of whom are self-taught—need to garner new skills to keep up with leading-edge technologies, or to reinforce knowledge they gained in the past. To remain current in their particular fields and able to develop new algorithms and tools in response to the continually shifting technological and computational sands, software developers must also have ongoing access to training programmes that address their advanced skill-development needs.

Against the backdrop of this fast-moving field, the challenges for bioinformatics education and training have increased, and a widening skills gap has been witnessed amongst life scientists [3–8]. Although rudimentary programming, use of bioinformatics tools and databases and statistical principles have appeared in some life science education programmes [9–11], basic data science is still relatively rare in bioscience curricula, creating a profound gap between theory and practice. In consequence, it is not uncommon for students to progress to research without adequate mathematical and computational foundations. This situation has generated significant concern, stimulating a range of surveys across academia, research institutes, professional bodies and industry, aiming to take the pulse of training needs worldwide. In the following pages, we discuss—at a purely qualitative level—the main conclusions from several of these surveys, and consider what progress has been made.

## The surveys

### SEB survey, 2013

In 2013, the Society for Experimental Biology (SEB) worked with the Global Organisation for Bioinformatics Learning, Education and Training (GOBLET) [12] to run a simple survey, aiming to capture a high-level picture of training needs within the SEB’s community of experimental biologists. To encourage participation, the questionnaire was deliberately brief: the questions focused on how bioinformatics training and education had been received; the level of confidence in using bioinformatics resources; how training should be delivered; and the skills most needed (Table 1).

**Table 1:** Questions used to survey the bioinformatics training needs of the SEB community

	Survey questions
1	What is your position/job title?
2	What is your main research discipline?
3	In which research institute/university/organization and in which country?
4	Which bioinformatics databases, software tools, analysis packages and/or interpretation techniques do you currently use in your research and for what purpose?
5	How confident are you using bioinformatics databases, tools, techniques, etc.?
6	How have you acquired bioinformatics knowledge/skills (past and present)?
7	Which bioinformatics skills training would you most value (e.g. database selection, software tools, analysing and interpreting data)?
8	How would you prefer training to be delivered to you?
9	Any other comments you would like to add?

The survey attracted ∼200 responses, from postgraduate students and technical staff, through to senior academics and managers. Of these, most (76%) considered their bioinformatics skills to be self-taught; only 20% reported having acquired their skills during university courses or programmes. In terms of their ability to use bioinformatics resources, 57% lacked confidence. Most respondents (67%) pointed to data analysis and interpretation as the greatest training needs, the majority (88%) preferring stand-alone workshops as the means of delivering bioinformatics training. Many also favoured access to online learning, and placed particular emphasis on the added value of tutor-supported options.

### GOBLET survey, 2014

Trends in these results were sufficiently interesting to prompt GOBLET to spread the questionnaire more widely through its partner organizations (in Europe, North America, Africa, Asia and Australia) to try to determine whether the findings were general across global communities of life and computational scientists. The wider survey attracted ∼500 responses from the full science career trajectory, and a range of roles and niches (i.e. the survey was not limited to wet-lab researchers but also acquired feedback from bioinformaticians, mathematicians, computer scientists, biophysicists and so on) [13, 14]. Of these, ∼64% considered themselves self-taught, having acquired their bioinformatics skills via peer support, online courses, professional workshops, etc.; 33% said they had received their training via university programmes; 57% lacked confidence in their ability to use bioinformatics resources. Many (54%) regarded statistics and data analysis/interpretation as their greatest training needs, the majority (65%) preferring stand-alone workshops and summer schools as the means of training delivery. University programmes were noticeably less popular (10%) as future routes for bioinformatics training.

### ABPI surveys, 2008 and 2014

During the past decade, the Association of the British Pharmaceutical Industry (ABPI), in collaboration with the BioIndustry Association (BIA), has also conducted surveys to try to understand current and future skill needs of the pharmaceutical industries, and to see how these are changing over time [6, 7]. Responses were solicited from a range of pharmaceutical companies, contract research organizations and small and medium enterprises. Interestingly, the major issues identified in 2008 included basic mathematical capability and the ability to apply scientific and mathematical knowledge. However, whilst a dearth of mathematical (especially statistical) skills was also highlighted in the 2014 survey, additional areas of concern relating to computational skills, including bioinformatics, data mining and health informatics (which involves use of electronic medical records, data mining, health app design and development, etc.), were not even raised as future concerns in 2008 [7].

### ELIXIR-UK industry survey, 2014

As part of its contribution to the ELIXIR infrastructure for life science information, ELIXIR-UK also conducted an industry survey (http://www.elixir-uk.org/industry-engagement), aiming to gain insights into training needs from an industrial perspective. Bioinformaticians and wet-lab scientists were targeted separately (71 and 86 respondents, respectively), with questions about their expertise in writing data analysis scripts, their degree of comfort with statistics, the skills they needed to be able to work more effectively and the preferred method of training delivery.

The analysis showed that 74% of wet-lab scientists had no programming experience (86% used Microsoft products like Excel for data analysis); 58% were not confident in their use of statistical methods (some were not even sure what statistical know-how they needed). This cohort considered expertise in data visualization (65%), statistics (61%) and data manipulation (58%) most important to acquire; 70% of bioinformaticians also sought training in statistics and data analysis methods. A majority of wet-lab scientists and bioinformaticians (61 and 51%, respectively) wanted their training to be delivered face-to-face, onsite, many also requesting online learning modules (43 and 56%, respectively). Most wet-lab scientists reported working with bioinformaticians, but 35% said they had no bioinformatician/statistician from whom to get support.

### NSF survey, 2016

In 2016, a needs assessment was conducted of principal investigators funded by the National Science Foundation (NSF) Directorate of Biological Sciences (DBS) [15]. The survey focused on 13 computational aspects of research (high-performance and cloud computing, workflows, analysis software, data storage, etc., Table 2), examining both infrastructural and training needs across research areas, research group sizes and DBS divisions (namely, Biological Infrastructure, Environmental Biology, Integrative Organismal Systems and Molecular and Cellular Biosciences).

**Table 2:** The 13 computational research and training needs investigated in the NSF survey

Computational research and training needs of NSF DBS investigators	
Research needs	Training and support needs
Publish data to the community	Data management and metadata
Sufficient data storage	Bioinformatics and data analysis
Share data with colleagues	Basic computing and scripting
Updated analysis software	Integration of multiple data types
Search for data and discover relevant data sets	Scaling analysis to cloud/HPC computing
Multistep analysis workflows or pipelines	
High-performance computing	
Cloud computing	

Of 704 respondents, 90% reported that they were (or would be within 3 years) analysing large data sets. Amongst their computational research and infrastructural needs, they confirmed that data publishing, data storage, data sharing and data analysis software were most important for their work. Interestingly, across all research disciplines, group sizes and BDS divisions, the greatest reported unmet needs were for training on integration of multiple data types (89%), data management and metadata (78%) and scaling analysis to cloud/High-Performance Computing (HPC) (71%).

### EMBL-ABR survey, 2016

In 2016, EMBL-Australia Bioinformatics Resource (ABR) reformulated the NSF survey, aiming to get an overview of the bioinformatics and computational biology research and training needs of life and medical scientists across Australia [16]. Amongst 123 respondents, the most important research needs were confirmed to be access to updated analysis software (91%), multistep analysis workflows/pipelines (82%) and high-performance/cluster computing (76%).

When specifically asked about training, the most pressing needs highlighted by respondents were in bioinformatics and analysis support (89%), basic programming and scripting (78%), data management and metadata (72%) and integrating multiple data types (67%). Fewer than 50% considered their training needs were being met by their institutional or national services.

## Understanding the training needs identified in the surveys

The proliferation of surveys bears witness to a growing concern about the computational and statistical competence of scientists across a range of disciplines, but especially in the life sciences. On one level, the narrative revealed by their collective results is straightforward. Nevertheless, these broad brushstrokes conceal a number of complex factors, the interplay between which will have influenced the survey responses, and must be considered when trying to understand the genuine training needs of specific audiences. For example, the time available for training is likely to have determined how individuals have received bioinformatics training in the past (whether focused and deep, broad and shallow, etc.); it is also likely to have shaped the level of confidence they have achieved in using bioinformatics tools and resources; it probably dictates what skills they still need (e.g. foundational or niche), and how they would prefer to acquire bioinformatics training in the future. Similarly, the background knowledge, profession and career level of individuals is likely to have determined their reasons for seeking training, how confident they are in using bioinformatics tools and resources, the skills they still need and their preferred format for future training.

Alongside factors that may have influenced individuals’ responses, it is also important to consider how the questions were phrased if we are to arrive at meaningful conclusions. For example, ∼75% of respondents to the GOBLET survey were beyond MSc/PhD stage [13, 14]. Hence, when asked, ‘How would you prefer training to be delivered to you?’, the responses were more likely to have favoured face-to-face courses/workshops and online resources because, in later career stages, university degree programmes are generally no longer the most relevant or practicable vehicles for learning. Phrased differently (e.g. ‘What is the most effective way for bioinformatics training to be delivered?’), the question might have elicited rather different answers. Not surprisingly, then, formal degree programmes emerged as the least popular routes for receiving future bioinformatics training, despite their obvious importance in providing fundamental bioinformatics education.

Equally important is what questions were asked. The questions in the SEB/GOBLET surveys were the same (albeit formatted slightly differently), but differed from those used in the NSF/EMBL-ABR surveys. In particular, questions in the former were largely open-ended, while those in the latter were leading, in the sense that responses were narrowed to a set of predefined research and training needs. In addition, the language used in the surveys was different: e.g. terms such as ‘data mining’, ‘data integration’, ‘data manipulation’, ‘data management’ and ‘metadata’ were used, but without a well-defined, shared vocabulary, these terms could have been interpreted differently by respondents.

## Take-home messages

With these caveats in mind, the survey results nevertheless highlight some consistency in the training gaps across the globe, and preferences for how bioinformatics training should be delivered to plug them. Collectively, the surveys highlight that training needs span a variety of topics. Amongst the needs considered most important were the acquisition of expertise and confidence in data/statistical analysis and interpretation. More broadly, skills in data management, data storage, data integration and data sharing (which might generally be grouped under the umbrella of ‘data stewardship’ or ‘data science’) were conspicuous needs in all the surveys conducted since 2013—some common themes are summarized in Table 3. Where preferences were expressed, short face-to-face courses and workshops were the most popular ways to receive training, alongside online resources.

**Table 3:** Summary of some of the most important training needs reported in recent surveys

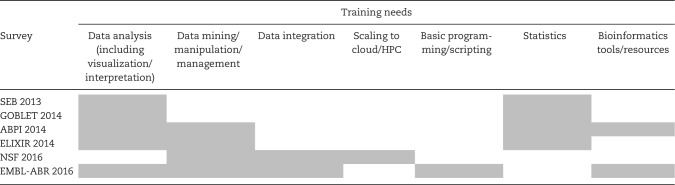

*Note*: Shaded cells denote needs identified by > 50% of respondents.

Perhaps the most urgent message, however, was that the next generation of life scientists should be reminded of the importance of bioinformatics and biostatistics throughout their studies, and that the learning process should commence with basic courses at undergraduate level, to introduce and instil the necessary skills at an early stage—ultimately, that bioinformatics should be fully integrated into life science degree programmes.

## The challenge for bioinformatics training

An important issue highlighted by the GOBLET survey was that most respondents had not gained their bioinformatics skills in university programmes [14]. Consequently, they had turned to peers, institutional or national services and/or short courses at the point of need. Being beyond the stage of formal university education, they preferred just-in-time training, especially via short online modules or face-to-face workshops, to help boost their confidence and to acquire new skills in bioinformatics and data stewardship.

Crucially, the need for bioinformatics training had only become apparent to some respondents after they had already designed their experiments and collected their data. This is both rather late in the research workflow, and suboptimal from a training perspective, because the foundational, broad-based bioinformatics education is not in place to build on, and skills acquired to address a specific need at a particular time are seldom retained. This engenders a cycle of low confidence in trainees, which is amplified if they cannot put their newly acquired skills into regular practice in the weeks and months since their training. It is therefore not uncommon for trainees to have to repeat courses, having forgotten what they learned previously [14].

The earlier computational thinking is introduced, and computational skills are embedded, the greater the likelihood of retention and sustained confidence in trainees. So, while there is still a strong demand for just-in-time training, in the longer term, investment in formal training at undergraduate level (if not sooner) is more likely to promote skill gain and retention [14]. Similar sentiments have been expressed by other commentators. Eddy [17], for example, suggested that all biologists should ‘learn to do their own data analysis’ and also learn ‘scripting [as] a fundamental lab skill, like pipetting’. While not all biologists need to master programming, incorporating bioinformatics earlier in the education cycle could help to bring more computationally minded biologists into wet-lab teams to help manage the programming and statistical components of data analyses [8, 14].

At the same time, the global bioinformatics training demand also argues for more and larger cohorts of individuals capable of delivering just-in-time training. Of course, there are already many established academic teachers with computational and/or statistical backgrounds. However, they will not always have experience in the required, niche-specific bioinformatics topics within their local communities. Moreover, while skilled in delivering traditional didactic coursework for semestered academic courses, they may struggle to adjust their content to short training programmes if they have not received guidance on how best to do so [18]. This situation warrants the development of programmes tailored for future trainers, to help disseminate best practices, to empower new bands of individuals to assuage local training needs and to ensure that trainers’ skills are kept up to date.

## Strengthening the global bioinformatics and data science training landscape

**Figure 1:**
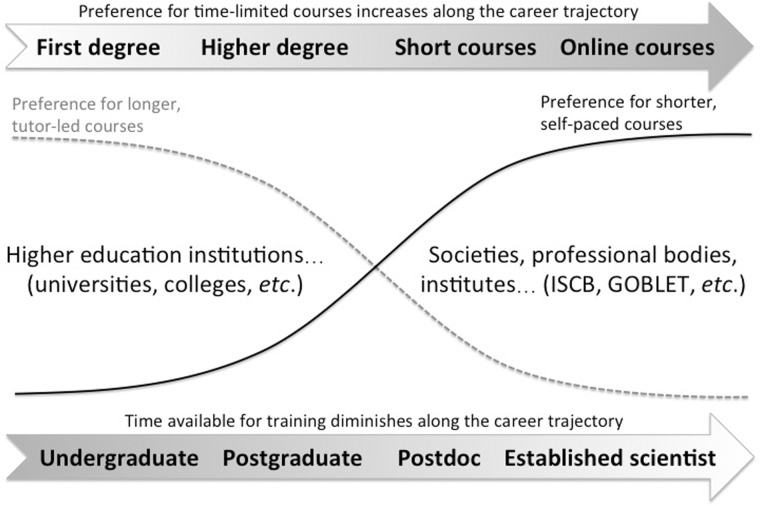
How trainees wish to acquire bioinformatics skills changes along the career trajectory. As the time available for training decreases (dotted line), individuals are more likely to move from semester-based academic programmes to shorter face-to-face and online courses that better serve the requisite just-in-time training needs (solid line).

Reviewing the outcomes of this diverse set of surveys from the past 5 years suggests that the need for bioinformatics and data science training is both real and urgent, and requires worldwide solutions. Short courses, whether delivered face-to-face or online (e.g. via MOOCs), have become the principal vehicles for plugging skill gaps, and their success (especially in areas such as next-generation sequencing data analysis, software and data ‘carpentry’ and so on) is a testament to the hard work and dedication of trainers around the globe. However, training provision is hampered both by the late stage at which many researchers seek training, and by the shortage of skilled trainers. Moreover, the ways in which individuals acquire, or wish to acquire, bioinformatics skills changes along their career paths, essentially in proportion to the amount of time they have available, as illustrated in Figure 1.

As highlighted in Figure 1, formal education and point-of-need training programmes provide complementary routes for the acquisition of bioinformatics and computational skills. Understanding the nature of this interplay should help educators and trainers to design courses and educational resources that better reflect the needs of trainees. For example, the format of current short courses could be revisited and the content segmented into smaller ‘bite-sized’ chunks for online delivery, to try to facilitate skill uptake and retention at the point-of-need.

Training portals [e.g. ELIXIR’s TeSS (http://tess.elixir-europe.org), GOBLET’s Training Portal (http://www.mygoblet.org) [19] and the Big Data to Knowledge (BD2K) Training Coordinating Center (TCC)’s Educational Resource Discovery Index, ERuDIte (http://bigdatau.org), to name just a few], which offer access to training materials in niche bioinformatics and data science topics, could provide better signposting to help guide trainees to the training formats and resources that would best meet their individual needs. Such portals could also be augmented with case studies and facilities to host discussion or ‘study groups’, where beginners could receive greater encouragement and peer support when learning how to use bioinformatics software tools and databases. Inclusion of short videos and quizzes/self-tests could also prove useful. In addition, creating post-course evaluation or certification methods could help to formalize and standardize the skills gain from, and the quality of, *ad hoc* training programmes.

The shortage of skilled point-of-need trainers has prompted initiatives and organizations like ELIXIR, GOBLET, H3ABionet (http://www.h3abionet.org) and EMBL-ABR (https://www.embl-abr.org.au) to begin developing and delivering specialized workshops for new and more experienced trainers (Train-the-Trainer workshops), covering not only the specific bioinformatics topics to be taught but also the principles important in providing robust training: how to define learning objectives, how to organize and set up short courses, how to develop and deliver materials, how to evaluate training results and so on [18, 20, 21]. These are aspects of teaching to which trainers might otherwise not be exposed; the Train-the-Trainer programme is thus developing a community of like-minded trainers with which to share experiences and to propagate such best practices and materials.

The dearth of computational skills allied to the data revolution has been recognised as an issue not just for life scientists but also for graduates in many scientific, medical and business-related disciplines. This has provided the impetus both for universities to develop new postgraduate programmes in generic data science, and for organizations like the Committee on Data for Science and Technology-Research Data Alliance (CODATA-RDA) to create research data-science summer schools (http://www.codata.org). It also saw the spin-out of Data Carpentry from the highly successful Software Carpentry Foundation (https://software-carpentry.org), offering training in core data skills to underpin reproducible research practices (http://www.datacarpentry.org).

## Discussion

Regardless of career position or role, skill gaps in computational aspects of biology impede the advancement of research and continue to fuel a global need for bioinformatics education and training. Looking back at the results of their 2005 survey, the ABPI warned, ‘It is quite clear that there has been little progress with many disciplines of critical concern to the pharmaceutical and biopharmaceutical industry. While timelines are short between the two surveys, time is also running out’ [6]. Yet, in 2015, they concluded that major mathematical and computational skills gaps were still causing significant problems with the number and quality of candidates available for recruitment [7]. These findings were echoed in the UK’s Biotechnology and Biological Sciences Research Council (BBSRC) and Medical Research Council (MRC) vulnerable skills report [22], which highlighted bioinformatics and data analytics (including programming and data stewardship) as particularly vulnerable in the biomedical sciences; skills in mathematics/statistics and computational biology were found to be especially lacking at postgraduate and postdoctoral levels, leading to recruitment problems with employers.

Observing the growing gap between the rapid accumulation of life science data and researchers’ ability to exploit these data effectively, Chang commented that the $215 million investment in the US Precision Medicine Initiative did not address ‘a worsening deficiency in the scientific community’ [5]. He argued that biologists should have more opportunities to learn bioinformatics skills, to increase the pool of applied bioinformaticians; otherwise, he warned, research will stall [5]. Similarly, Barone *et al.* [15] opined, ‘This portends a growing data knowledge gap in biology and challenges institutions and funding agencies to redouble their support for computational training in biology’.

Such is the scale and urgency of currently unmet training needs that it is tempting to consider it a new phenomenon, but this is not the case. Even in the 1990s, the supply of bioinformaticians and computer scientists could not meet the demand for individuals able to analyse and manage the then large quantities of data (which today, in the era of second- and third-generation sequencing technologies, seem trivial by comparison). In light of advancing genomics research, a BBSRC review suggested a need to increase bioinformatics staff by 60% in academia and by 45% in pharmaceutical companies (similar conclusions were drawn in Canada [23] and elsewhere). At the time, the shortage of skilled individuals stimulated a much-needed Research Council investment in studentships, senior fellowships, summer schools and so on [24]; MacLean and Miles (1999) observed that similar responses had been made by funding agencies in France, Germany, Switzerland, Australia and the United States. While progress was being made in bioinformatics training provision, however, they urged that ‘swift action [was] needed to close the skills gap’ [24] because the growing quantities of data could only fully be exploited with the application of bioinformatics skills.

Brass cautioned that such investment left no room for complacency [25]. Amongst the many hurdles ahead were the difficulty of teaching bioinformatics at undergraduate and postgraduate levels (students often leaving higher-degree courses with only superficial mathematical and computational skills), and the difficulty of finding suitably qualified candidates for lectureship posts. Nevertheless, funding was not sustained and the problems persisted. By 2008, the ABPI suggested that although some progress had been made to enhance the skills and capabilities of students aged 14–19 years, the response from funders of higher-education programmes had been ‘patchy’ [6]. In 2010, Cummings and Temple [3] continued to emphasize the need for bioinformatics training across the entire spectrum, from novice biologists to expert practitioners; however, Sarkar acknowledged that ‘acquiring bioinformatics competency can become a complex endeavor of undecipherable jargon, mathematics and frustration’ [26].

Lamenting the lack of integration of bioinformatics into senior high-school curricula in 2013, Machluf and Yarden [27] argued that it is ‘playing almost no role in preparing the next generation of information-oriented citizens’. They contended that bioinformatics education must evolve in line with advances in bioinformatics research, informed by dialogues at the interface of bioinformatics curriculum design and teaching and learning processes [27]. Similarly, to address the demand for training, Carvalho and Rustici [4] called for new paradigms in bioinformatics education, both to train scientists to become trainers and to develop new teaching resources (including e-learning courses).

Despite the ‘swift action’ urged by MacLean and Miles almost 20 years ago, the bioinformatics skills gap has not narrowed; in fact, the training deficit is set to widen as the data science revolution takes hold, bringing with it new training imperatives for life scientists of tomorrow. Attempting to tackle this problem, international organizations like GOBLET, ELIXIR, the International Society for Computational Biology (especially via its Computational Biology Education Community of Special Interest; https://www.iscb.org/iscb-cosis), CODATA-RDA, EMBL-ABR and BD2K TCC are increasing their efforts to enlarge the community of trainers and make more bioinformatics training resources available. However, the independent surveys reviewed here suggest that the grass-roots efforts of societies and professional bodies are not likely to be enough. Policy makers and funders must also heed the warning signs and join the discussion on how to ensure that continued bioinformatics skill gaps do not create insurmountable barriers to the progress of research. Universities must continue to strive to bring life science curricula into the digital data era [14]; but even before students reach university, high schools must also start to play their part to increase the mathematical and computational proficiency of aspiring life scientists. To support the necessary changes to high-school and university education programmes, and to nurture new cohorts of trainer scientists, sustained investment is required—short-term funding bursts, like those witnessed in the 1990s, are not sufficient. Ultimately, lecturers, teachers, trainers, policy makers and funding agencies across the world must work together to turn the dream of ‘swift action’ into a tangible, deliverable action plan to quench the global thirst for bioinformatics training.

##

**Key Points**

- Surveys of global communities of life and computational scientists in the past 5 years show a widening bioinformatics skills gap—the training deficit has not improved over the past 20 years.
- In later career stages, stand-alone workshops and online resources are favoured routes for acquiring training at the point of need.
- To improve skill retention and confidence, bioinformatics should be embedded early, as a core component of life science degree programmes.
- More programmes are needed to train future trainers, to help disseminate best practices and keep trainers’ skills up to date.
- A concerted, worldwide response is required from all stakeholders to deliver a tangible action plan, with sustained investment, to transform high-school and university programmes and cultivate a new cadre of trainer scientists.

## References

[1] Tan TW, Lim SJ, Khan AM, et al. A proposed minimum skill set for university graduates to meet the informatics needs and challenges of the “-omics” era. BMC Genomics 2009;10(Suppl 3):S36. 19958501 10.1186/1471-2164-10-S3-S36 PMC2788390

[2] Welch L, Lewitter F, Schwartz R, et al. Bioinformatics curriculum guidelines: toward a definition of core competencies. PLoS Comput Biol 2014;10:e1003496. 24603430 10.1371/journal.pcbi.1003496 PMC3945096

[3] Cummings MP, Temple GG. Broader incorporation of bioinformatics in education: opportunities and challenges. Brief Bioinform 2010;11:537–43. 20798182 10.1093/bib/bbq058

[4] Carvalho BS, Rustici G. The challenges of delivering bioinformatics training in the analysis of high-throughput data. Brief Bioinform 2013;14:538–47. 23543353 10.1093/bib/bbt018 PMC3771233

[5] Chang J. Core services: reward bioinformaticians. Nature 2015;520:151–2. 25855439 10.1038/520151a

[6] ABPI. Skills needs for biomedical research. Creating the pool of talent to win the innovation race. http://www.abpi.org.uk/our-work/mandi/Documents/2008-10%20STEM%20Skills%20Review%20Report%20FINAL%20amended2.pdf#search=skills%2520need%2520for%2520biomedical%2520research (28 April 2017, date last accessed).

[7] ABPI. Bridging the skills gap in the biopharmaceutical industry. http://www.abpi.org.uk/our-work/library/industry/Documents/Skills_Gap_Industry.pdf (28 April 2017, date last accessed).

[8] Brazas MD, Blackford S, Attwood TK. Training: plug gap in essential bioinformatics skills. Nature 2017;544:161. 10.1038/544161c 28406196

[9] Goodman AL, Dekhtyar A. Teaching bioinformatics in concert. PLoS Comput Biol 2014;10:e1003896. 25411792 10.1371/journal.pcbi.1003896 PMC4238947

[10] Libeskind-Hadas R, Bush E. A first course in computing with applications to biology. Brief Bioinform 2013;14:610–17. 23449003 10.1093/bib/bbt005

[11] Rubinstein A, Chor B. Computational thinking in life science education. PLoS Comput Biol 2014;10:e1003897. 25411839 10.1371/journal.pcbi.1003897 PMC4238948

[12] Attwood TK, Bongcam-Rudloff E, Brazas MD, et al. GOBLET: The Global Organisation for Bioinformatics Learning, Education and Training. PLoS Comput Biol 2015;11:e1004143. 25856076 10.1371/journal.pcbi.1004143 PMC4391932

[13] SEB/GOBLET Bioinformatics Workshop: An essential tool for experimental biologists. http://www.mygoblet.org/sites/default/files/goblet_events/SEBGOBLETWorkshopReport070714.pdf (28 April 2017, date last accessed).

[14] Brazas MD, Brooksbank C, Jimenez RC, et al. A global perspective on bioinformatics training needs. BioRXiv 2017. doi: 10.1101/098996.

[15] Barone L, Williams J, Micklos D. Unmet needs for analysing biological big data: a survey of 704 NSF principal investigators. BioRXiv 2016. doi: 10.1101/108555. PMC5654259 29049281

[16] Schneider MV, Flannery M, Griffin P. Survey of bioinformatics and computational needs in Australia 2016. figshare, 2016 10.6084/m9.figshare.4307768.v1 (28 April 2017, date last accessed).

[17] Eddy S. High throughput sequencing for neuroscience, 2014 Cryptogenomicon on WordPress.com. http://cryptogenomicon.org/2014/11/01/high-throughput- sequencing-for-neuroscience/ (28 April 2017, date last accessed).

[18] Via A, Blicher T, Bongcam-Rudloff E, et al. Best practices in bioinformatics training for life scientists. Brief Bioinform 2013;14:528–37. 23803301 10.1093/bib/bbt043 PMC3771230

[19] Corpas M, Jimenez RC, Bongcam-Rudloff E, et al. The GOBLET training portal: a global repository of bioinformatics training materials, courses and trainers. Bioinformatics 2015;31:140–2. 25189782 10.1093/bioinformatics/btu601 PMC4271145

[20] Schneider MV, Watson J, Attwood T, et al. Bioinformatics training: a review of challenges, actions and support requirements. Brief Bioinform 2010;11:544–51. 20562256 10.1093/bib/bbq021

[21] Watson-Haigh NS, Shang CA, Haimel M, et al. Next-generation sequencing: a challenge to meet the increasing demand for training workshops in Australia. Brief Bioinform 2013;14:563–74. 23543352 10.1093/bib/bbt022 PMC3771231

[22] BBSRC and MRC vulnerable skills report. 2015 http://www.bbsrc.ac.uk/about/policies/reviews/consultations/1501-vulnerable-capabilities-report/ (28 April 2017, date last accessed).

[23] White paper: Bioinformatics curriculum recommendations for undergraduate, graduate and professional programs, July 25, 2002 https://www.biotalent.ca/sites/biotalent/files/PDF/BioinformaticsWhitePaper.pdf (28 April 2017, date last accessed).

[24] MacLean M, Miles C. Swift action needed to close the skills gap in bioinformatics. Nature 1999;401:10. 10.1038/43269 10485694

[25] Brass A. Bioinformatics education—a UK perspective. Bioinformatics 2000;16:77–8. 10842726 10.1093/bioinformatics/16.2.77

[26] Sarkar IN. Editorial: bioinformatics education in the 21st century. Brief Bioinform 2010;11:535–6. 21087950 10.1093/bib/bbq071

[27] Machluf Y, Yarden A. Integrating bioinformatics into senior high school: design principles and implications. Brief Bioinform 2013;14:648–60. 23665511 10.1093/bib/bbt030

